# Gray and White Matter Volumes and Cognitive Dysfunction in Drug-Naïve Newly Diagnosed Pediatric Epilepsy

**DOI:** 10.1155/2015/923861

**Published:** 2015-08-31

**Authors:** Jung Hwa Lee, Song E. Kim, Chang-hyun Park, Jeong Hyun Yoo, Hyang Woon Lee

**Affiliations:** ^1^Department of Neurology, Ewha Womans University School of Medicine and Ewha Medical Research Institute, Seoul 158-710, Republic of Korea; ^2^Department of Neurology, Samsung Changwon Hospital, Sungkyunkwan University School of Medicine, Changwon 630-723, Republic of Korea; ^3^Department of Radiology, Ewha Womans University School of Medicine and Ewha Medical Research Institute, Seoul 158-710, Republic of Korea

## Abstract

Epilepsy patients often have cognitive dysfunction even at early stages of disease. We investigated the relationship between structural findings and neuropsychological status in drug-naïve newly diagnosed pediatric epilepsy patients. Thirty newly diagnosed pediatric epilepsy patients and 25 healthy control subjects aged 7~16 years were enrolled, who were assessed by the Korean version of the Wechsler Intelligence Scale for Children (K-WISC-III), the Stroop test, and the trail making test (TMT). Optimized voxel-based morphometry (VBM) was performed for both Gray Matter (GM) and White Matter (WM) volumes. Lower performance levels of verbal intelligence quotient, freedom from distractibility, and executive function were observed in epilepsy group. Interestingly, poor performance in these cognitive subdomains was correlated with regional VBM findings involving both GM and WM volumes, but with different patterns between groups. GM volumes revealed clear differences predominantly in the bilateral frontal regions. These findings indicate that certain cognitive functions may be affected in the early stage of epilepsy, not related to the long-standing epilepsy or medication, but more related to the neurocognitive developmental process in this age. Epilepsy can lead to neuroanatomical alterations in both GM and WM, which may affect cognitive functions, during early stages even before commencement of AED medication.

## 1. Introduction

Cognitive or intellectual function is an important issue in childhood epilepsy since epilepsy can have significant effects on the development and function of the immature brain. It has been reported that cognitive abilities, particularly verbal and nonverbal intelligence quotients (IQ) and executive function, are impaired in childhood epilepsy [1, 2]. Previous studies reported that cognitive impairments in epilepsy patients are related to various clinical factors such as the age at onset [1], longer duration or type of seizures [2], epileptic syndrome [3], and use of antiepileptic drugs (AEDs) [4]. However, a small number of studies showed that children with newly diagnosed epilepsy also have neuropsychological dysfunctions [5–8]. To obtain a clear perspective of the potential progressive and lifetime neuropsychological consequences of epilepsy, it is important to characterize cognitive impairments in pediatric patients with new-onset epilepsy before the AED medication. Interestingly, each epileptic syndrome tends to be associated with specific cognitive dysfunctions in children with new-onset epilepsy [7], for example, language dysfunction in benign childhood epilepsy with centrotemporal spikes (BECTS) [9], memory impairment in temporal lobe epilepsy [10], attention deficit disorder in absence epilepsy [11], and executive dysfunction in juvenile myoclonic epilepsy [12].

Many studies using quantitative analyses of structural magnetic resonance imaging (MRI), such as volumetry, cortical thickness, and/or diffusion tensor imaging (DTI), have suggested structural brain abnormalities as the etiology of cognitive impairment in childhood epilepsy. Volumetric studies on pediatric epilepsy revealed abnormalities in the cerebrum [13], cerebellum, and hippocampus [14, 15] as well as temporal and extratemporal Gray Matter (GM) [16, 17]. In children and adolescents with epilepsy, cognitive and behavioral problems are significantly correlated with decreased cortical thickness in specific brain regions [18]. A recent study using DTI showed that White Matter (WM) abnormalities in the dominant frontal and temporal regions were related to language, executive function, and intelligence in BECTS [9]. Quantitative MR volumetry has been used to characterize the nature and pattern of brain abnormalities in adults with epilepsy, especially temporal lobe epilepsy, and volumetric abnormalities are one of the clinical consequences, as demonstrated by their relationship with impaired cognition [19–24]. However, there have been only a few volumetric studies conducted in children [6, 14, 15, 25]. Although there have been several studies in children and adolescents with epilepsy associated with neuropsychological abnormalities [6], the neuroanatomical basis is not fully understood yet. Examination of children at the time of epilepsy onset would help elucidate the neuroanatomical correlates of cognitive dysfunction in pediatric epilepsy. This study was performed to further extend this line of research by investigating (i) the neuropsychological status, especially intellectual ability and executive function before AED medication, (ii) regional GM and WM volumes in the epileptic brains, and (iii) association patterns between regional GM/WM volumes and cognitive function in patients with newly diagnosed pediatric epilepsy before AED administration.

## 2. Materials and Methods

### 2.1. Subjects

The study population consisted of 30 children and adolescents with drug-naïve newly diagnosed pediatric epilepsy. The inclusion criteria were (1) newly diagnosed epilepsy with no AED administration prior to participation; (2) seizure onset within 12 months based on history; (3) age between 7 and 16 years; and (4) attendance at standard schools. Diagnosis of epilepsy was made by expert neurologists (Jung Hwa Lee and Hyang Woon Lee) based on clinical, electroencephalographic (EEG), and MRI findings. The diagnosis of epileptic syndrome was based on the International League Against Epilepsy criteria [26]. Exclusion criteria were (1) developmental disabilities; (2) IQ <70; and (3) any history of usage of medications acting on the central nervous system.

The control group included 25 normal children who were close friends of the index patients and were similar in age, gender, socioeconomic status, and educational level. Children with IQ <70, a history of usage of medications acting on the central nervous system, no attendance at standard schools, a past or current history of neuropsychiatric disorders or head injury, or a first-degree relative with a history of epilepsy or febrile convulsions were excluded.

The patients with drug-naïve newly diagnosed epilepsy included 16 males and 14 females, with a mean age of 10.5±2.9 years (Table 1). Of the 25 control subjects, 13 were male and 12 female, with a mean age of 11.1±3.0 years. The average durations of education were 4.6±3.1 and 4.9±3.2 years in the patient and control groups, respectively. Twenty-one patients (21/30, 70%) were diagnosed as focal epilepsy, and the remaining nine patients (30%) were diagnosed as generalized epilepsy.

Structural MRI and neuropsychological evaluations were performed in the patients at the time of diagnosis before commencement of AED medication. Signed informed consent was obtained for all participants from their parents or guardians. This study was approved by the Human Investigation Committee of the Ewha Womans University Medical Center.

### 2.2. Neuropsychological Assessment

All subjects underwent a comprehensive neuropsychological testing battery including (1) the Korean version of Wechsler Intelligence Scale for Children-III (K-WISC-III) standardized for Korean children and adolescents, consisting of 10 standard and 3 supplementary subsets, including full scale IQ, verbal IQ, and performance IQ, verbal comprehension (information, similarities, vocabulary, and comprehension), perceptual organization (picture completion, picture arrangement, block design, and object assembly), freedom from distractibility (coding, symbol search), and processing speed (coding and symbol search) [27, 28]; (2) the Stroop-color-word association test [29]; and (3) the trail making test (TMT) A and TMT B for Children [30]. Raw test scores were converted to age-adjusted scores. The reliability of the K-WISC-III was reported with Cronbach's alpha coefficients of 0.84, 0.92, and 0.68 for full scale IQ, verbal IQ, and performance IQ, respectively [28].

### 2.3. MRI Acquisition

MRI examinations were performed using a 3 T scanner (Philips Achieva v2.6, Best, Netherlands) in all subjects. Whole-brain 3D T1-weighted gradient echo images were acquired for each subject using the magnetization-prepared rapid acquisition with gradient echo (MPRAGE) sequence (TR = 1160 ms, TE = 4.19 ms, TI = 600 ms, field of view = 140×250 mm^2^, matrix size = 256×192, slice thickness = 1.2 mm, and flip angle = 15°), yielding 130~140 contiguous coronal slices depending on the head size with a defined voxel size of 0.94×0.94×1.2 mm^3^. Together with the volumetric data, T1-weighted axial, T2-weighted axial/oblique coronal, and FLAIR axial/oblique coronal images were also acquired as part of an epilepsy MRI protocol (5 mm thickness for each sequence).

### 2.4. Voxel-Based Morphometry

Optimized voxel-based morphometry (VBM) was used for quantitative analysis of MRI, similarly to previous studies [31, 32] using SPM8 (Wellcome Trust Centre for Neuroimaging, http://www.fil.ion.ucl.ac.uk) implemented in MATLAB 7.3 (The MathWorks, Natick, MA, USA). Following the standard protocol, VBM analysis was performed in the following order [33–35].

Since we included subjects with wide range of age, we created a customized template appropriate to the population samples [34, 35]. Each image was spatially normalized to the standard MNI template included in SPM8. The normalized image was then smoothed with a 6-mm full width at half maximum (FWHM) Gaussian kernel, and a mean image was created as the study-specific template.

For spatial normalization, all images in native space were transformed to the same stereotactic space by registering each to the template image. We followed the normalization procedure in SPM8 using default options. That is, the affine registration that determined the optimum 12 parameters was followed by estimating nonlinear deformations defined by a linear combination of three-dimensional discrete cosine transform [36]. According to the default options, each of the deformation fields was described by 1176 parameters that represented coefficients of the deformations in three orthogonal directions. The spatially normalized images were resliced to a final voxel size of 1 mm^3^ to yield more accurate subsequent tissue segmentation [37].

The normalized images were then segmented into GM, WM, and CSF. With a mixture model clustering algorithm, voxel intensities matching particular tissue types were identified. The segmentation step also incorporated correction for image intensity nonuniformity [38].

Finally, the normalized, segmented images were smoothed using a 10-mm FWHM Gaussian kernel. This made the images conform more closely to the Gaussian random field model [38], which supported inferences about regionally specific effects in subsequent statistical analysis.

### 2.5. Statistical Analysis

The neuropsychological test scores were compared between the patient and control groups after adjustment for age and gender using multivariate analysis of covariance (MANCOVA). Education level was not adjusted since all of the subjects in both patient and control groups were enrolled for Korean standard education system and the duration of education years did not show any difference between the groups. Wilcoxon's signed rank test was used for comparison of the focal and generalized epilepsy groups. We also examined other clinical factors including EEG focus/lateralization, age at onset, type of seizures, and seizure frequency for their effects on cognitive functions using ANOVA. Statistical analyses were performed using SPSS version 12.0 (SPSS Inc., Chicago, IL, USA). In all analyses, *P* < 0.05 was taken to indicate statistical significance, and *F* values with Cohen's *d* distributions were presented for each cognitive domain.

For statistical analysis of VBM, analysis of covariance (ANCOVA) was applied for voxelwise comparison of GM and WM volumes between the epilepsy and control groups, with age and gender as confounding covariates. The false discovery rate (FDR) was applied to correct for multiple comparisons at *P* < 0.05 [39] and the contiguous voxel extent threshold was set to 100 voxels. The anatomical localization of significant clusters was identified using Talairach coordinates [40].

To investigate the relationship between VBM GM/WM volumes and psychological performance scores, a general linear model was applied. First, the values of each voxel in GM and WM were extracted separately as the “volume of interest” (VOI), without designating a priori assumption by choosing specific brain regions, and then correlated with cognitive test scores, using partial correlation coefficients with correction for age and gender, in the epilepsy and control groups, respectively. For group differences from VBM, age and gender were adjusted as covariates of no interest, and FDR correction with the same extent threshold of 100 contiguous voxels was used for statistical significance (*P* < 0.05).

## 3. Results

### 3.1. Analysis of Neuropsychological Tests

Table 2 provides a comparison of neuropsychological performance between the control and epilepsy groups. The epilepsy group had lower verbal IQ and freedom from distractibility mean scores, as well as longer response times in the Stroop-color and Stroop-color-word tests compared with controls (*P* < 0.05). There were similar trends of decreased scores in most cognitive subdomains especially verbal comprehension, perceptual organization, or processing speed. To determine whether there were distinctive neuropsychological performance patterns between the different epileptic syndromes, focal and generalized epilepsy groups were also compared. Patients with focal epilepsy showed lower verbal IQ scores compared with generalized epilepsy patients. Similarly, perceptual organization tended to be lower in focal epilepsy than generalized epilepsy group. There were no other significant differences in each cognitive domain between the different epileptic syndromes (Table 3). There were no significant associations in other clinical factors including EEG focus/lateralization, age at onset, type of seizures, and epileptic syndrome.

### 3.2. Analysis of Voxel-Based Morphometry

**Figure 1:**
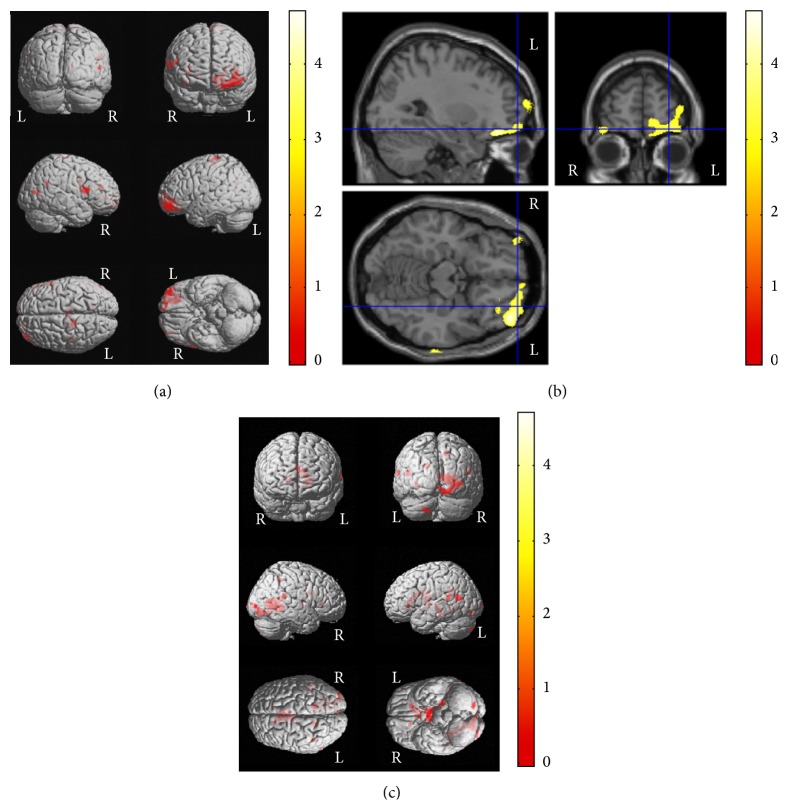
VBM results of brain regional differences between patients with newly diagnosed pediatric epilepsy and control subjects and correlations between VBM results and cognitive functions in control subjects. Patients with newly diagnosed pediatric epilepsy showed VBM abnormalities in both GM and WM areas compared with control subjects at the time of diagnosis, which suggests these could be more related to the epilepsy itself rather than long-standing seizures or medication effects. GM volumes were decreased in the left inferior frontal and right middle frontal gyri in epilepsy patients compared with control subjects ((a) and (b)). In addition, association patterns between structural findings and cognitive functions showed clear difference between groups; that is, decreased verbal IQ scores were correlated with decreased GM volumes in the left superior temporal and anterior cingulate gyri and decreased WM volumes in the left superior temporal and the right parahippocampal gyri in control subjects (c), but no correlation was observed in epilepsy patients. Please also note Tables 4 and 5 for *x*, *y*, *z* coordinates and more detailed information. L, left; R, right.

Optimized VBM analysis was performed in 30 epilepsy patients and 25 control subjects to compare cerebral GM and WM volumes. Most prominently, GM volume was decreased in the left inferior frontal and right middle frontal gyri of the patients compared with the controls (corrected *P* < 0.001) (Figures 1(a) and 1(b)). Detailed *x*, *y*, *z* coordinates and more information were summarized in Table 4.

### 3.3. Relationships between Cognition and VBM Findings

In whole-brain voxel correlation analysis using three cognitive variables with significant group differences, lower verbal IQ scores were correlated with decreased GM volumes in the left superior temporal and anterior cingulate gyri and decreased WM volumes in the left superior temporal and the right parahippocampal gyri in the control group (corrected *P* < 0.001) (Figure 1(c)). However, no correlations were found between the verbal IQ score and GM/WM volumes in the epilepsy group. Detailed *x*, *y*, *z* coordinates and more information were summarized in Table 5.

**Figure 2:**
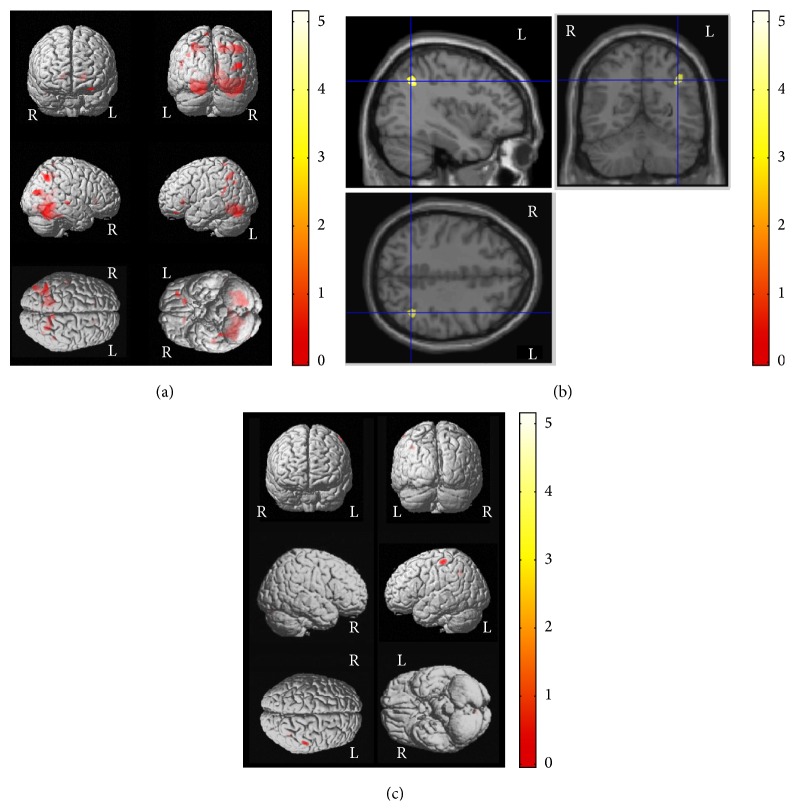
Correlations between VBM results and cognitive functions in patients with newly diagnosed pediatric epilepsy and control subjects. Association patterns between structural findings and cognitive functions showed some differences between the groups. Lower scores in freedom from distractibility were correlated with decreased WM volumes in the right parahippocampal and the middle temporal gyri and the left frontal subgyral, precuneus, and the superior parietal lobule areas in the control group (corrected *P* < 0.001) (a). However, lower scores in freedom from distractibility were correlated with decreased GM volumes in the left postcentral gyrus in the epilepsy group (corrected *P* = 0.033) ((b) and (c)). Please also note Table 5 for *x*, *y*, *z* coordinates and more detailed information. L, left; R, right.

In addition, lower scores in freedom from distractibility were correlated with decreased WM volumes in the left frontal subgyral area, precuneus, and the superior parietal lobule as well as the right parahippocampal and middle temporal gyri in the control group (corrected *P* < 0.05) (Figure 2(a) and Table 5). However, lower scores in freedom from distractibility were correlated with decreased GM volumes in the left postcentral gyrus in the epilepsy group (corrected *P* = 0.033) (Figures 2(b), 2(c) and Table 5).

**Figure 3:**
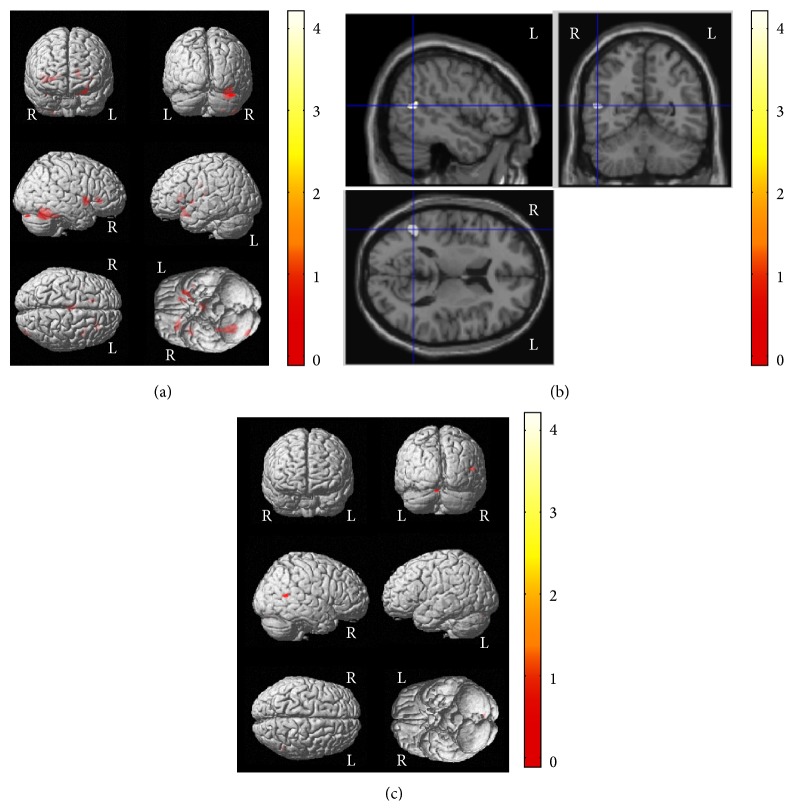
Correlations between VBM results and cognitive functions in patients with newly diagnosed pediatric epilepsy and control subjects. Association patterns between structural findings and cognitive functions showed clear differences between the groups. In the control group, poor performance in the Stroop-color test (longer response time) was correlated with decreased GM volume in the right posterior lobe and decreased WM volumes of the right frontal subgyral and insular areas and the left sublobar region (a). In contrast, in the epilepsy group, a longer response time in the Stroop-color test was correlated with decreased GM volume only of the right superior temporal gyrus in the epilepsy group ((b) and (c)). Please also note Table 5 for *x*, *y*, *z* coordinates and more detailed information. L, left; R, right.

A longer response time in the Stroop-color test was correlated with decreased GM volume of the right posterior lobe and decreased WM volumes of the right frontal subgyral and insular areas and part of the left sublobar region in the control group (corrected *P* < 0.01) (Figure 3(a) and Table 5). In contrast, a correlation between Stroop-color test response time and GM volume was observed only in the right superior temporal gyrus in the epilepsy group (corrected *P* = 0.033) (Figures 3(b), 3(c) and Table 5). No significant correlations were observed for other cognitive variables in either the control or the epilepsy group.

## 4. Discussion

Using neuropsychological tests and VBM, we investigated the neuropsychological status and structural brain alterations in children and adolescents with drug-naïve newly diagnosed epilepsy. In addition, we characterized the relationship between each cognitive domain and specific brain region. Patients with newly diagnosed pediatric epilepsy showed (i) poor performance in several cognitive domains, verbal IQ, freedom from distractibility scores, and response time of the Stroop-color test; (ii) decreased GM volume in the left inferior frontal and right middle frontal gyri; and (iii) distinct association patterns between structural findings and cognitive functions for freedom from distractibility and Stroop test performance compared with those in control subjects. These findings suggest that cognitive functions, especially verbal IQ, freedom from distractibility, and executive function, could be affected in pediatric epilepsy patients at the time of diagnosis, not related to the long-standing epilepsy and/or AED medication effects but more related to the early insult from epilepsy itself in the rapidly growing young brain, especially in the frontal regions.

As we mentioned already, this study was performed in drug-naïve childhood and adolescents with newly diagnosed epilepsy before they started AED medication. Similar to previous studies in new-onset epilepsy [6, 8], neuropsychological dysfunction in several cognitive subdomains and executive function in newly diagnosed epilepsy patients in the present study cannot be explained by disease duration or AEDs, as all evaluations were performed before commencement of AED administration. These findings support previous reports that the neuropsychological status in children and adolescents with epilepsy seems to be affected at a very early stage of the disease, even before AED treatment. In fact, most cognitive domains showed similar tendency of impairment in epilepsy patients compared with control subjects, including significant differences in verbal IQ, freedom from distractibility, Stroop test, and marginal significance in verbal comprehension, perceptual organization, and processing speed. In subgroup analyses, patients with focal epilepsy showed lower verbal IQ scores and similar trend of lower perceptual organization score compared with those with generalized epilepsy. One possible reason for this may be that the majority of focal epilepsy was BECTS, which is reportedly related to early involvement of language dysfunction mainly affecting the dominant hemisphere [9, 41].

In this study, we also investigated the possible associations between cognitive dysfunctions and regional GM/WM volumes in patients with newly diagnosed pediatric epilepsy. In the present study, the most prominent structural abnormalities observed in newly diagnosed pediatric epilepsy were decreased GM volumes in the bilateral frontal areas, especially the left inferior frontal and right middle frontal gyri. Based on the results of this study and previous VBM studies in pediatric epilepsy, predominant frontal structural abnormalities may be a common pattern of pediatric epilepsy across various epileptic syndromes. Several possible mechanisms may account for why these abnormalities are observed mainly in the frontal area. First, direct tissue damage due to recurrent epileptic discharges may be possible since the frontal lobe occupies the largest volume of the brain. Second, cortical damage may result from an interaction between brain development and seizures. The areas most vulnerable to damage could be those that are not only connected to an epileptogenic region, but also undergoing a period of rapid development [42]. Different brain areas undergo peaks in growth and maturation at different stages [43], and frontal regions are also known to undergo marked structural changes, especially in cortical thickness during adolescence as a result of pruning. As this study included adolescents, the frontal lobe may be the most vulnerable area affected by seizures. This would require further validation, possibly via longitudinal studies in patients of a more restricted age range.

Several studies indicated significant correlations between general intelligence and regional GM density or volume [44, 45]. In the present study, poor performance in several cognitive subdomains was correlated with regional VBM abnormalities with clear differences between the control and epilepsy groups. Lower scores in freedom from distractibility were correlated with decreased WM volumes of the left frontal subgyral area, precuneus, and superior parietal lobule and the right parahippocampal and middle temporal gyri in the control group, whereas the correlation was only observed in the GM volume of the left postcentral gyrus in the epilepsy group. In addition, longer response time in the Stroop-color test was correlated with decreased GM volume in the right posterior lobe and decreased WM volumes especially in the right frontal subgyral and insular regions in the control group. In contrast, this kind of correlation was observed only in GM volume of the right superior temporal gyrus in the epilepsy patients. These findings suggested potential early involvement of their cognitive dysfunction associated with neuroanatomical abnormalities developing early in the course of disease progression in newly diagnosed pediatric epilepsy.

The present study had several limitations. Differences in various epileptic syndromes could not be detected as the numbers of patients with each syndrome were insufficient for separate analyses. Future long-term prospective investigations with larger patient populations and individualized studies in each epileptic syndrome are required. This study focused mainly on intellectual ability and executive function due to time restrictions, since we aimed to perform all investigations before commencement of AEDs. All patients in the epilepsy group were attending standard schools in order to compare them with control subjects of similar educational levels. Patients who are not attending school are also worth evaluating, as they may have higher rates of various behavioral and academic problems and/or learning disabilities. Further investigations of newly diagnosed epilepsy patients with distinct epilepsy syndromes including careful prospective study designs with longitudinal follow-up of the same patient groups would be beneficial to identify more clear understanding of neuropsychological, behavioral, academic/learning problems of these patients. Another promising approach could be more advanced imaging methods accompanied by technical improvement by means of the advanced image registration algorithm, for instance, tensor-based morphometry using DARTEL algorithm [46] for more sensitive detection of subtle and early neuroanatomical alterations in future longitudinal studies.

## 5. Conclusion

In summary, we evaluated neuropsychological performance and relationships with respect to GM and WM volumes demonstrated by optimized VBM analysis in children and adolescents with newly diagnosed epilepsy. Pediatric epilepsy patients showed poorer performance in verbal IQ, freedom from distractibility, and executive function at the time of epilepsy diagnosis before commencement of AEDs. GM volumes revealed clear differences predominantly in the bilateral frontal regions. These findings indicate that cognitive functions, especially verbal IQ and executive function, may be affected in the early stage of epilepsy, not related to the long-standing epilepsy or AED medication but more related to the neurocognitive developmental process in these young pediatric age groups.

## Figures and Tables

**Table 1:** Demographic data of the epilepsy and control groups.

Patient number	Epilepsy group		Control group
	Focal epilepsy	Generalized epilepsy	
Number of patients	21	9	25
Age (years)	10.5±2.9		11.1±3.0
Gender (M : F)	16 : 14		13 : 12
Education duration	4.6±3.1 years		4.9±3.2 years
Duration of epilepsy	3.4±2.5 months		
Epileptic syndromes	BECTS 13 BCEOP 2 FLE 4 TLE 2	JME 5 CAE 2 JAE 1 IGE 1	

M: male; F: female; BECTS: benign childhood epilepsy with centrotemporal spikes, BCEOP: benign childhood epilepsy with occipital paroxysm, FLE: frontal lobe epilepsy, TLE: temporal lobe epilepsy, JME: juvenile myoclonic epilepsy, CAE: childhood absence epilepsy; JAE, juvenile absence epilepsy, IGE: idiopathic generalized epilepsy. Data are shown as means±standard deviation.

**Table 2:** Comparison of neuropsychological performance between control and epilepsy patient groups.

Cognitive test domain	Control	Epilepsy	*F *	Cohen's *d*	*P* value
Full scale IQ	110.05 ± 18.3	106.82 ± 16.72	2.038	0.184	0.160
Verbal IQ	106.1 ± 15.77	101.39 ± 15.57	4.138	0.301	0.048^*∗*^
Performance IQ	109.38 ± 18.27	105.12 ± 16.93	3.286	0.242	0.076
Verbal comprehension	109.05 ± 18.15	105.27 ± 16.78	3.183	0.216	0.081
Perceptual organization	108.1 ± 14.76	104.06 ± 15.07	3.602	0.271	0.064
Freedom from distractibility	112.1 ± 18.92	107.57 ± 17.2	3.946	0.249	0.047^*∗*^
Processing speed	99.05 ± 15.96	94.27 ± 14.8	3.434	0.311	0.070
Stroop-color (RT, s)	13.7 ± 9.6	27.4 ± 17.5	6.071	0.947	0.018^*∗*^
Stroop-color (no. of errors)	0.35 ± 0.59	0.48 ± 0.77	0.534	0.190	0.469
Stroop-cw (RT, s)	24.4 ± 15.13	27.38 ± 17.47	0.033	0.182	0.857
Stroop-cw (no. of errors)	0.59 ± 0.83	1.21 ± 1.77	3.205	0.513	0.081
TMT-A (RT, s)	24.85 ± 10.11	30.02 ± 14.17	1.224	0.420	0.276
TMT-B (RT, s)	71.85 ± 32.43	92.79 ± 60.81	1.166	0.430	0.287

IQ: intelligence quotient, Stroop-cw: Stroop-color-word test, RT: response time, s: seconds, no.: number, and TMT: trail making test, data shown as mean ± standard deviation, ^*∗*^ *P* < 0.05 by MANCOVA.

**Table 3:** Comparison of neuropsychological performance between generalized and focal epilepsy groups.

Cognitive test domain	Generalized epilepsy	Focal epilepsy	*F *	Cohen's *d*	*P* value
Full scale IQ	103.78 ± 17.97	104.57 ± 15.44	0.004	0.047	0.952
Verbal IQ	105.89 ± 17.05	98.1 ± 14.81	4.593	0.488	0.042^*∗*^
Performance IQ	105.22 ± 19.04	102.13 ± 15.54	0.753	0.178	0.394
Verbal comprehension	100.89 ± 19.37	102.63 ± 15.51	0.079	0.099	0.781
Perceptual organization	108.11 ± 16.68	101.23 ± 14.88	3.945	0.435	0.058
Freedom from distractibility	101 ± 14.07	104.4 ± 15.44	0.348	0.230	0.561
Processing speed	95.11 ± 10.87	90.93 ± 13.19	0.968	0.346	0.334
Stroop-color (RT, s)	16.2 ± 6.16	15.98 ± 5.43	2.584	0.038	0.125
Stroop-color (errors)	0.35 ± 0.59	0.48 ± 0.77	0.294	0.190	0.594
Stroop-cw (RT, s)	24.4 ± 15.13	27.38 ± 17.47	1.022	0.182	0.325
Stroop-cw (errors)	0.5 ± 0.83	1.21 ± 1.77	1.753	0.514	0.202
TMT-A (RT, s)	24.85 ± 10.11	30.02 ± 14.17	0.054	0.420	0.818
TMT-B (RT, s)	71.85 ± 32.43	92.79 ± 60.81	2.277	0.430	0.149

IQ: intelligence quotient, Stroop-cw: Stroop-color-word test, RT: response time, s: seconds, no.: number, and TMT: trail making test, data shown as mean ± standard deviation, ^*∗*^ *P* < 0.05 by MANCOVA.

**Table 4:** Brain regions that showed group differences between the control and epilepsy groups for Figures 1(a) and 1(b).

Group difference	Anatomical regions	Coordinate			Cluster size	Cluster-level corrected *P* value
		(*x*	*y*	*z*)		
Control > Epilepsy	Left inferior frontal gyrus, Gray Matter	−17	13	21	5261	<0.001
	Right middle frontal gyrus, Gray Matter	26	40	−19	2191	<0.001

Control > Epilepsy means the brain areas that showed more decreased VBM values in the epilepsy group compared with those in the control group.

**Table 5:** Brain regions correlated with intelligence scores in the control and epilepsy groups for Figures 1(c), 2, and 3.

Group	Cognitive subdomain	Anatomical regions	Coordinate			Cluster size	Cluster-level corrected *P* value
			(*x*	*y*	*z*)		
Control	Verbal IQ	Left superior temporal gyrus, Gray Matter	−67	−62	14	1983	<0.001
		Left anterior cingulate gyrus, Gray Matter	−46	−44	15	721	<0.001
		Left superior temporal gyrus, White Matter	−16	−44	26	934	<0.001
		Right parahippocampal gyrus, White Matter	32	−48	−3	13973	<0.001

Control	Freedom from distractibility	Left frontal lobe, Subgyral, White Matter	−17	28	−2	851	<0.001
		Left parietal lobe, precuneus, White Matter	17	−58	42	4952	0.016
		Left superior parietal lobule, White Matter	−28	−55	45	2208	<0.001
		Right parahippocampal gyrus, White Matter	32	−51	−6	36365	<0.001
		Right middle temporal gyrus, White Matter	38	−72	19	2015	<0.001

Control	Stroop-color	Right posterior lobe, Gray Matter	33	−59	−27	4779	<0.001
		Right frontal lobe, Subgyral, White Matter	23	30	0	1034	0.005
		Right insular, White Matter	40	8	−2	1360	0.001
		Left cerebrum, Sublobar, White Matter	−2	−13	15	2472	<0.001

Epilepsy	Freedom from distractibility	Left postcentral gyrus, Gray Matter	−53	−28	60	766	0.033

Epilepsy	Stroop-color	Right superior temporal gyrus, Gray Matter	19	−54	12	475	0.033

IQ: intelligence quotient.

## References

[1] Elger C. E., Helmstaedter C., Kurthen M. (2004). Chronic epilepsy and cognition. *The Lancet Neurology*.

[2] Vingerhoets G. (2006). Cognitive effects of seizures. *Seizure*.

[3] Tromp S. C., Weber J. W., Aldenkamp A. P., Arends J., vander Linden I., Diepman L. (2003). Relative influence of epileptic seizures and of epilepsy syndrome on cognitive function. *Journal of Child Neurology*.

[4] Rzezak P., Fuentes D., Guimarães C. A. (2007). Frontal lobe dysfunction in children with temporal lobe epilepsy. *Pediatric Neurology*.

[5] Fastenau P. S., Johnson C. S., Perkins S. M. (2009). Neuropsychological status at seizure onset in children: risk factors for early cognitive deficits. *Neurology*.

[6] Hermann B., Jones J., Sheth R., Dow C., Koehn M., Seidenberg M. (2006). Children with new-onset epilepsy: neuropsychological status and brain structure. *Brain*.

[7] Hermann B. P., Jones J. E., Jackson D. C., Seidenberg M. (2012). Starting at the beginning: the neuropsychological status of children with new-onset epilepsies. *Epileptic Disorders*.

[8] Oostrom K. J., Smeets-Schouten A., Kruitwagen C. L. J. J., Peters A. C. B., Jennekens-Schinkel A. (2003). Not only a matter of epilepsy: early problems of cognition and behavior in children with ‘epilepsy only’—a prospective, longitudinal, controlled study starting at diagnosis. *Pediatrics*.

[9] Kim S. E., Lee J. H., Chung H. K., Lim S. M., Lee H. W. (2014). Alterations in white matter microstructures and cognitive dysfunctions in benign childhood epilepsy with centrotemporal spikes. *European Journal of Neurology*.

[10] Bell B., Lin J. J., Seidenberg M., Hermann B. (2011). The neurobiology of cognitive disorders in temporal lobe epilepsy. *Nature Reviews Neurology*.

[11] Caplan R., Siddarth P., Stahl L. (2008). Childhood absence epilepsy: behavioral, cognitive, and linguistic comorbidities. *Epilepsia*.

[12] Pascalicchio T. F., Filho G. M. D. A., Noffs M. H. D. S. (2007). Neuropsychological profile of patients with juvenile myoclonic epilepsy: a controlled study of 50 patients. *Epilepsy & Behavior*.

[13] Hermann B. P., Dabbs K., Becker T. (2010). Brain development in children with new onset epilepsy: a prospective controlled cohort investigation. *Epilepsia*.

[14] Lawson J. A., Vogrin S., Bleasel A. F. (2000). Predictors of hippocampal, cerebral, and cerebellar volume reduction in childhood epilepsy. *Epilepsia*.

[15] Lawson J. A., Cook M. J., Vogrin S. (2002). Clinical, EEG, and quantitative MRI differences in pediatric frontal and temporal lobe epilepsy. *Neurology*.

[16] Cormack F., Gadian D. G., Vargha-Khadem F., Cross J. H., Connelly A., Baldeweg T. (2005). Extra-hippocampal grey matter density abnormalities in paediatric mesial temporal sclerosis. *NeuroImage*.

[17] Dabbs K., Jones J. E., Jackson D. C., Seidenberg M., Hermann B. P. (2013). Patterns of cortical thickness and the Child Behavior Checklist in childhood epilepsy. *Epilepsy and Behavior*.

[18] Baxendale S. A., van Paesschen W., Thompson P. J. (1998). The relationship between quantitative MRI and neuropsychological functioning in temporal lobe epilepsy. *Epilepsia*.

[19] Bernasconi N., Duchesne S., Janke A., Lerch J., Collins D. L., Bernasconi A. (2004). Whole-brain voxel-based statistical analysis of gray matter and white matter in temporal lobe epilepsy. *NeuroImage*.

[20] Dow C., Seidenberg M., Hermann B. (2004). Relationship between information processing speed in temporal lobe epilepsy and white matter volume. *Epilepsy and Behavior*.

[21] Griffith H. R., Pyzalski R. W., Seidenberg M., Hermann B. P. (2004). Memory relationships between MRI volumes and resting PET metabolism of medial temporal lobe structures. *Epilepsy and Behavior*.

[22] Hermann B., Seidenberg M., Sears L. (2004). Cerebellar atrophy in temporal lobe epilepsy affects procedural memory. *Neurology*.

[23] Martin R. C., Hugg J. W., Roth D. L. (1999). MRI extrahippocampal volumes and visual memory: correlations independent of MRI hippocampal volumes in temporal lobe epilepsy patients. *Journal of the International Neuropsychological Society*.

[24] Seidenberg M., Kelly K. G., Parrish J. (2005). Ipsilateral and contralateral MRI volumetric abnormalities in chronic unilateral temporal lobe epilepsy and their clinical correlates. *Epilepsia*.

[25] Lawson J. A., Cook M. J., Bleasel A. F., Nayanar V., Morris K. F., Bye A. M. E. (1997). Quantitative MRI in outpatient childhood epilepsy. *Epilepsia*.

[26] Engel J. (2001). A proposed diagnostic scheme for people with epileptic seizures and with epilepsy: report of the ILAE task force on classification and terminology. *Epilepsia*.

[27] Park K. W., Yoon J. R., Park H. J., Kim K. W. (1991). *Manual for the Korean Education Development Institute-Wechsler Intelligence Scale for Children*.

[28] Kwak K. J., Park H. W., Kim C. T. (2002). The standardization study for the Korean Wechsler intelligence scale for children—third edition (1): reliability and validity. *Korean Journal of Developmental Psychology*.

[29] Shin M. S., Park M., Stroop J. (2007). *Stroop Color and Word Test: A Manual for Clinical and Experimental Uses*.

[30] Lee J. B., Kim J. S., Seo W. S., Shin H. J., Bai D. S., Lee H. L. (2003). The validity and reliability of computerized neurocognitive function test in the elementary school child. *Korean Journal of Psychosomatic Medicine*.

[31] Good C. D., Johnsrude I. S., Ashburner J., Henson R. N. A., Friston K. J., Frackowiak R. S. J. (2001). A voxel-based morphometric study of ageing in 465 normal adult human brains. *NeuroImage*.

[32] Ridgway G. R., Henley S. M. D., Rohrer J. D., Scahill R. I., Warren J. D., Fox N. C. (2008). Ten simple rules for reporting voxel-based morphometry studies. *NeuroImage*.

[33] Keller S. S., Wilke M., Wieshmann U. C., Sluming V. A., Roberts N. (2004). Comparison of standard and optimized voxel-based morphometry for analysis of brain changes associated with temporal lobe epilepsy. *NeuroImage*.

[34] Yoon U., Fonov V. S., Perusse D., Evans A. C. (2009). The effect of template choice on morphometric analysis of pediatric brain data. *NeuroImage*.

[35] Wilke M., Schmithorst V. J., Holland S. K. (2002). Assessment of spatial normalization of whole-brain magnetic resonance images in children. *Human Brain Mapping*.

[36] Ashburner J., Friston K. J. (2000). Voxel-based morphometry—the methods. *NeuroImage*.

[37] Kim J. H., Lee J. K., Koh S.-B. (2007). Regional grey matter abnormalities in juvenile myoclonic epilepsy: a voxel-based morphometry study. *NeuroImage*.

[38] Worsley K. J., Andermann M., Koulis T., MacDonald D., Evans A. C. (1999). Detecting changes in nonisotropic images. *Human Brain Mapping*.

[39] Genovese C. R., Lazar N. A., Nichols T. (2002). Thresholding of statistical maps in functional neuroimaging using the false discovery rate. *NeuroImage*.

[40] Lancaster J. L., Woldorff M. G., Parsons L. M. (2000). Automated Talairach atlas labels for functional brain mapping. *Human Brain Mapping*.

[41] Verrotti A., D'Egidio C., Agostinelli S., Parisi P., Chiarelli F., Coppola G. (2011). Cognitive and linguistic abnormalities in benign childhood epilepsy with centrotemporal spikes. *Acta Paediatrica, International Journal of Paediatrics*.

[42] Holmes G. L., Ben-Ari Y., Zipursky A. (2001). The neurobiology and consequences of epilepsy in the developing brain. *Pediatric Research*.

[43] Gogtay N., Giedd J. N., Lusk L. (2004). Dynamic mapping of human cortical development during childhood through early adulthood. *Proceedings of the National Academy of Sciences of the United States of America*.

[44] Frangou S., Chitins X., Williams S. C. R. (2004). Mapping IQ and gray matter density in healthy young people. *NeuroImage*.

[45] Wilke M., Sohn J.-H., Byars A. W., Holland S. K. (2003). Bright spots: correlations of gray matter volume with IQ in a normal pediatric population. *NeuroImage*.

[46] Ashburner J. (2007). A fast diffeomorphic image registration algorithm. *NeuroImage*.

